# Effects of work conditions on provider mental well-being and quality of care: a mixed-methods intervention study in the emergency department

**DOI:** 10.1186/s12873-018-0218-x

**Published:** 2019-01-03

**Authors:** Anna Schneider, Markus Wehler, Matthias Weigl

**Affiliations:** 1 Institute and Clinic for Occupational, Social, and Environmental Medicine, University Hospital, LMU Munich, Munich, Germany; 2 Department of Emergency Medicine and Department of Medicine IV, Klinikum Augsburg, Augsburg, Germany

**Keywords:** Work conditions, Emergency department, Nurses, Physicians, Mental well-being, Intervention, Quality of care, Patient survey, Emergency care, Emergency service, Hospital

## Abstract

**Background:**

Emergency departments (EDs) are highly dynamic and stressful care environments that affect provider and patient outcomes. Yet, effective interventions are missing. This study evaluated prospective effects of a multi-professional organizational-level intervention on changes in ED providers’ work conditions and well-being (primary outcomes) and patient-perceived quality of ED care (secondary outcome).

**Methods:**

A before and after study including an interrupted time-series (ITS) design over 1 year was established in the multidisciplinary ED of a tertiary referral hospital in Southern Germany. Our mixed-methods approach included standardized provider surveys, expert work observations, patient surveys, and register data. Stakeholder interviews were conducted for qualitative process evaluation. ITS data was available for 20 days pre- and post-intervention (Dec15/Jan16; Dec16/Jan17). The intervention comprised ten multi-professional meetings in which ED physicians and nurses developed solutions to work stressors in a systematic moderated process. Most solutions were consecutively implemented. Changes in study outcomes were assessed with paired t-tests and segmented regression analyses controlling for daily ED workload.

**Results:**

One hundred forty-nine surveys were returned at baseline and follow-up (response at baseline: 76 out of 170; follow-up: 73 out of 157). Forty-one ED providers participated in both waves. One hundred sixty expert work observations comprising 240 observation hours were conducted with 156 subsequent work stress reports. One thousand four hundred eighteen ED patients were surveyed. Considering primary outcomes, respondents reported more job control and less overtime hours at follow-up. Social support, job satisfaction, and depersonalization deteriorated while respondents’ turnover intentions and inter-professional interruptions increased. Considering the secondary outcome, patient reports indicated improvements in ED organization and waiting times. Interviews revealed facilitators (e.g., comprehensive approach, employee participation) and barriers (e.g., understaffing, organizational constraints) for intervention implementation.

**Conclusions:**

To the best of our knowledge, this is the first study to report prospective effects of an ED work system intervention on provider well-being and patient-perceived quality of ED care. We found inconsistent results with partial improvements in work conditions and patient perceptions of care. However, aspects of provider mental well-being deteriorated. Given the lack of organizational-level intervention research in EDs, our findings provide valuable insights into the feasibility and effects of participatory interventions in this highly dynamic hospital setting.

**Electronic supplementary material:**

The online version of this article (10.1186/s12873-018-0218-x) contains supplementary material, which is available to authorized users.

## Background

Emergency departments (EDs) are highly dynamic work environments with particular risks for provider well-being and quality of care [1]. Adverse work factors contribute to high work stress among ED providers [2]. Burnout has been reported by 26% of emergency nurses [3] and by up to 51% of emergency physicians [4]. Suboptimal patient care was also linked to adverse ED work factors such as poor teamwork or frequent workflow interruptions [5–7]. Notwithstanding the need for effective interventions concerning ED work conditions, there is a dearth of reported interventions [1, 2, 8]. Thus far, research on prospective interventions targeting psychosocial ED work factors is limited [2, 4, 8, 9]: First, an intervention study with ED nurses in three Chinese emergency care facilities showed that comprehensive management (nurse manager-led meetings on communication skills, conflicts, efficacy elevation, and emotion control) was related to lower burnout levels over 6 months [10]. Second, a teamwork intervention in California at four ED sites was associated with improved perceptions of the ED work environment among ED nurses and physicians [11]. In this study, EDs participated in a teamwork training curriculum (Emergency Team Coordination Course) where teamwork principles such as maintaining team structure and climate, problem solving strategies, team communication, executing plans and managing workload as well as team skills were practiced. Lastly, an 18-month prospective study of nurses in 15 Belgian EDs observed that changes in work conditions, such as job demands, control, social support, reward, social harassment and work agreements, were associated with job satisfaction, work engagement, emotional exhaustion and turnover intentions [12]. These study findings indicate that job demands were relatively stable whereas social support and material resources showed most variation over time; turnover intentions deteriorated [12]. Yet this observational research omitted any specification of actual intervention measures. Overall, available studies have shortcomings such as lack of theoretical foundation, insufficient methodological rigor for identification of prospective effects, and sole focus on specific ED professions [2, 8].

The Systems Engineering Initiative for Patient Safety (SEIPS) framework [13] provides a sociotechnical model of interdependencies between several work factors, provider and patient outcomes in healthcare [13]. The underlying premise is that multiple factors (tasks, technologies, persons, environment, organization) conjointly generate stress loads and affect provider and organizational outcomes, i.e., provider health as well as patient safety [14]. Furthermore, participative interventions apply systematic analyses as well as collaborative improvement of work conditions [15–17]. Thus ‘front-line’ providers, such as ED physicians and nurses, are best suited to identify and solve problems in their work environment [18, 19]. Although preliminary evidence suggests that organizational-level interventions can be effective in eliciting positive changes in work systems and provider well-being, thorough evaluations concerning intervention effects on quality of care outcomes are scarce [20].

The aim of our study was to investigate the effects of ED work system factors on provider well-being and quality of ED care, using the SEIPS model as a theoretical framework. Second, we set out to evaluate the feasibility and effectiveness of a multi-professional organizational-level intervention with focus on both ED provider and patient outcomes. Third, we used mixed-methods interrupted time-series (ITS) evaluations to determine intervention effects. An ITS paradigm is recommended as a quasi-experimental surrogate for assessing intervention effectiveness when a randomized controlled trial is not feasible [21, 22].

## Methods

A prospective intervention study with a mixed-methods ITS design encompassing a 12-month observation period was established. Methods included (1) standardized provider surveys, (2) structured work observation sessions with ED physicians and nurses and concurrent work stress reports, (3) patient surveys, (4) register data, and (5) stakeholder interviews, which are described below. Observation sessions, work stress reports, and patient surveys were conducted on-site on 20 days each at baseline (Dec 15 – Jan 16) and follow-up (Dec 16 – Jan 17), respectively (exact dates are listed in Additional file 1: Table S1). ED providers were informed about the study via presentations and information leaflets. Written informed consent was obtained prior to data collection. The Ethics Committee of the Medical Faculty, Munich University, approved the study (NR 327–15).

### Setting and sample

The study setting is a 24-h interdisciplinary ED of a tertiary referral hospital in a major city in Southern Germany. The academic hospital provides major services and medical specialties for an administrative region of almost two million inhabitants. The ED serves adult patients with mean yearly visits of over 85,000. It is organized in three sections according to patient’s chief complaints: i.e., ten separate bays for non-surgical patients, five separate bays for surgical patients, two resuscitation bays, and an observation and clinical decision unit with 24 beds. The ED is regularly staffed with junior and senior physicians from internal medicine, trauma surgery, and neurology, as well as further specialists on call.

The study team approached two hospitals for participation, whereof this ED’s department head, hospital administration, and hospital’s worker council agreed to take part. The study team established first contact directly with the head of the ED. The head discussed the proposal with the head of nursing and introduced it in two team meetings of ED physicians and nurses, respectively. After positive feedback from ED staff, the study was presented at the hospital board meeting and received approval. The specific motivation and decision for participation were not specified prior to study start. However, the ED was described as a high strain work environment with particular challenges for nurses, physicians, and patient care. All professionals working in the ED, i.e., nurses, physicians, and administrative staff, were eligible for participation. At baseline, ED staff consisted of 101 nurses (including assistant nurses), 44 physicians, and 20 administrators. External providers and on-call consultants were not included.

### Methods and study outcomes

Proposed intervention effects on (a) work system factors, (b) provider mental well-being, and (c) quality of care were identified using the following methods:

1. Provider survey

ED providers received surveys at baseline and follow-up that were distributed through internal mail. Pre-stamped envelopes were provided for direct return of questionnaires to the study team. Deadline for survey completion was four weeks and estimated average time for filling in questionnaires was 25 min. Matching across time was ensured through personalized study codes.

(1a) *Work system factors* were measured with a validated self-report tool for work analysis in hospitals that has been previously applied to ED work settings [23, 24]. Following SEIPS framework, several work factors were surveyed. Task-related factors included scales on patient stressors (i.e., dealing with difficult patients; three items), job control (i.e., autonomous decision making and personal discretion; four items), participation opportunities (i.e., influence on work-related decisions; four items), work overload (i.e., job duties exceeding work time; three items). Organizational factors consisted of personnel resources (i.e., adequate staffing; three items), information problems (i.e., availability and clarity of work-related information; three items), uncertainty (i.e., job insecurity; three items), social support (i.e., support by colleagues and supervisors; three items), and supervisor feedback (i.e., feedback on performance and task behaviors by senior leaders; two items). Self-reported mean weekly overtime (in hours and minutes), profession (ED physician, ED nurse, ED administrator) and professional tenure (in years) were further retrieved.

(1b) *Provider well-being* included two key burnout dimensions, i.e., emotional exhaustion and depersonalization (four items each) [9] and a screening tool for depressive symptoms (two items) [25]. Both tools are validated and have been previously applied in healthcare as well as ED provider samples [8, 24]. Conventional cut-off criteria were used to determine providers with elevated levels of emotional exhaustion (scale mean > 3.5) [26] and depressive symptoms (scale sum score ≥ 3) [25]. Job satisfaction and turnover intentions were measured with one item, respectively.

(1c) Provider perceptions of *quality of care* were measured with a three-item scale on the frequency of medical errors [27]. Respondents were asked to indicate whether they had experienced a near miss, minor error, or serious error during the past year. For each type of error, a short definition was provided. Additionally, overall patient safety was further assessed with one item (“Please rate the degree of patient safety in your department from your point of view”) [28].

(2). Work observation sessions and work stress reports

Observation sessions of ED nurses’ and physicians’ workflows were allocated randomly across three ED sections and professions. Randomization and sessions were limited to provider day shifts between 10:00 am and 5:00 pm on 40 pre-defined days of data collection. Trained observers shadowed providers for 90-min sessions using a standardized participant observation approach that has been previously applied to ED settings [6, 29].

(2a) *Work system factors* were represented by observed interruption rates. An established tool to identify workflow interruptions was applied [6, 29]. Referencing the SEIPS model, we distinguished between interruptions initiated by patients and their relatives (task-related factors), and those by ED colleagues of the same or another profession (organizational factors). Furthermore, duration of personal breaks (e.g., time for personal rests, short respites from work, or regular pauses during the shift) during observation sessions was coded (in % of observed time) [30].

(2b) *Provider well-being* was surveyed immediately after each observation session with a short survey on current cognitive, emotional, and physical aspects of work stress in each observed provider [31].

(3). Patient survey

All ED patients undergoing consultation or treatment on days of on-site data collection were eligible. After information and verbal consent, patients filled in the survey. Patients’ relatives were allowed to fill in the survey by proxy if they accompanied patients throughout their ED stay. Patients with incapability to communicate due to illness severity or other physical and mental constraints were not surveyed. The patient questionnaire was handed out by members of the study team, preferably at the end of patients’ ED treatment. All study team members received prior training in how to approach and interview ED patients.

(3c) Patient perceptions of ED *quality of care* were obtained with a standardized patient survey (Munich Patient Inventory) with additional translations in English, Russian, and Turkish language. This questionnaire assesses patient-perceived quality of care [32] and has been tested for reliability and validity in different clinical settings [33, 34], including the ED setting [6]. It contains scales on the quality of interaction (example item: “My problems and complaints are taken seriously by ED providers”), patient information (e.g., “I am comprehensively informed about the course of therapy and treatment”), ED organization (e.g., “I know who of the ED providers is responsible for me”), and waiting time (e.g., “My waiting time until the first consultation with an ED physician was adequate”). Additionally, patients’ overall satisfaction with care was obtained with one question (“Overall, how do you evaluate care in this ED?”).

(4). Register data on ED workload

An approximate measure for daily ED workload was computed with day-level data on patient numbers, patient acuity (ESI, Emergency Severity Index score), and staffing levels. Data was extracted from ED administrative records and staff rosters.

(5). Stakeholder interviews

To complement quantitative results, we used ED stakeholder interviews for qualitative process evaluation [35, 36]. Nine stakeholder interviews were conducted at study half-time and follow-up. A semi-structured interview guideline assessed intervention implementation, facilitators and barriers, and providers’ mental models [35]. Questions were derived from Nielsen & Randell (2013)‘s framework for comprehensive process evaluation of organizational-level interventions [35]. The German interview guideline can be obtained from the corresponding author. Interviewees were recruited through convenience sampling. All interviewed stakeholders had a job tenure > 5 years in the ED, except one junior physician. Four physicians (three with leadership responsibilities), four nurses (one in leadership position) and one ED administrator were interviewed. Overall, there were four female and five male interviewees. The department head, nursing supervisor, and administration head were interviewed twice to comprise intervention implementation at ED management level at half-time as well as follow-up. Interviews lasted between 30 and 60 min. We did not apply a prior estimate of expected sample size for data saturation since project resources did not allow for more stakeholder interviews.

### Intervention

The intervention started after feedback of baseline survey results to ED staff during regular internal meetings as well as through internal mail. Ten 90-min meetings, termed health circles [15], were held at three-week intervals over a period of seven months. Three to seven ED nurses and physicians participated in each meeting. Principally, all ED providers were invited to take part. Decision for participation was completely at the discretion of each ED provider. Practically, the majority of participants consisted of staff members who were on duty on days of respective meetings; for nurses, usually before or after their shift; for physicians, mostly in breaks during their shift. Therefore, participants varied considerably across individual meetings. Participation in health circles was considered work time. Additionally, ED nursing management and the hospitals’ workers council (German: ‘Personalrat’; employee representatives being elected by hospital employees) attended. All meetings were moderated by the study team. In the first meeting, the concept of health circles was introduced. Potential advantages of this approach (i.e., tailored to local needs, employee involvement, intervention process adapted to context) as well as potential problems (e.g., insufficient implementation of solutions, organizational constraints, time-consuming and long-lasting process of structural re-organization) were discussed. Afterwards, participants identified adverse ED work conditions in a systematic process facilitated by moderators: Participants classified problematic work conditions according to their practical importance and potential for change and formed an agenda of issues for improvement for subsequent meetings. Each of the following meetings focused on the development of measures for one of the identified work problems. In these guided health circles, participants collectively analyzed and discussed potential solutions for work problems and defined an action plan with concrete measures, persons responsible, and deadlines [19]. Two meetings were designated to evaluate implementation status of measures and to discuss potential adjustments. Each health circle meeting was documented in written form and made available to the entire ED staff through the intranet information system. Additionally, participating nurses and physicians were assigned to report on health circle meeting outcomes in their regular team meetings.

After each health circle meeting, participants provided short feedback on their satisfaction with and effectiveness of the respective meeting. We used a self-developed tool that measured five aspects: (1) participant’s satisfaction with meeting outputs (“I am satisfied with the results of today’s health circle”), (2) meeting atmosphere (“I am satisfied with today’s group atmosphere”), (3) opportunities to actively develop solutions for work problems (“In this health circle I can actively contribute to improvements of our work situation”), (4) motivation to improve work conditions (“Today’s health circle meeting motivates me to improve processes and contents of my work”), and (5) participant’s expectations of actual implementation of developed solutions in everyday work (“The developed solutions can be implemented in our daily workflow”). All questions were answered on five-point Likert-Scale ranging from 1=“no, not at all” to “5 = yes, exactly”.

In addition to health circles, three meetings of a steering committee were held during the intervention period. These meetings comprised ED management (ED head, head nurse), the hospitals’ work council and health promotion department, and head of ED administration. The steering committee discussed measures developed by ED staff which could not be implemented immediately, needed approval from ED management, or affected inter-departmental or hospital-wide coordination and decisions [15]. In each meeting, the committee reviewed the action plan, monitored project status, and discussed measures developed in health circle meetings with regard to their implementation in routine work organization and processes.

### Analyses

First, to identify proposed intervention effects, changes in provider survey results between baseline and follow-up were calculated using paired t-tests. To assess strength of changes, Cohen’s effect sizes were calculated and classified as weak (0.2–0.4), moderate (0.5–0.7), or strong effects (> 0.8) [37]. Second, ITS data of hourly interruption rates from work observation sessions, work stress reports, and patient perceptions of care were aggregated to mean scores at the day-level. Data was analyzed with segmented regression analysis with 40 available data points, i.e., 20 each pre- and post-intervention [21]. A daily ED workload measure was calculated from mean daily staffing levels and number and acuity of patients as indicated by ESI scores [38]. Autoregressive integrated moving average (ARIMA) models were estimated and controlled for ED workload [39]. In all steps, listwise deletion was used for missing data. All quantitative analyses were conducted with SPSS 24 (IBM, Chicago)**.** Interview data was analyzed applying content analysis. All nine interviews were audio-taped and transcribed verbatim to cluster recurrent main themes [40].

## Results

### Intervention implementation

In the first health circle meeting, participants identified six adverse ED work conditions: (1) lack of personal breaks (i.e., regular work breaks and personal pauses were often omitted due to high workload); (2) work agreements (i.e., unspecified agreements on patient care and tasks that hinder fast and efficient care); (3) high work pressure environment: point of triage (i.e., ED’s location where incoming patients are assessed for the severity of their symptoms based on a standardized process), (4) leadership, (5) staff information (i.e., insufficient information on current reorganizational projects), and (6) staff shortages (i.e., sustained understaffing of ED personnel). Thirteen respective measures were developed focusing on improvements in task-related and organizational work factors (see Table 1). Implementation fidelity varied by the time of follow-up: Eight solutions were implemented or in progress. Five were deemed unfeasible by the steering committee due to financial constraints and personnel shortages and were not pursued.

**Table 1:** Action plan of ED providers’ identified issues for improvement, respective measures, and implementation status at follow-up

Work system factor (SEIPS)	Identified problems and issues for improvement	Solutions and respective improvement measures	#HCM / #SCM	Implementation status at follow-up
Organization	Lack of personal breaks during work time (i.e., limited opportunities to take breaks while on duty; short duration of breaks; multiple short breaks instead of longer pauses)	Schedule additional nursing staff for short-term replacement of nurses taking breaks	HCM#2 / SCM#2, SCM#4	Not feasible and declined after discussion in steering committee
		Short-term rotation across ED units to replace nursing staff in breaks	HCM#2/ SCM#2, SCM#4	Partially completed
		Supervising physicians coordinate residents’ breaks	HCM#2/ SCM#2	Fully completed
		Shift supervisor coordinates temporary replacement of nursing staff in breaks on a daily basis	HCM#2/ SCM#2, SCM#4	Declined after discussion in steering committee
Task	Unclear work agreements (i.e., lack of mutual agreement between ED units concerning patient transfers and admissions; unclear agreements with ICU and adjacent care units concerning specific care obligations, e.g., timing of transfusions)	Revise agreements for interdisciplinary occupancy of ED observation unit	HCM#3 / SCM#2, SCM#4	Discussed with consulting physicians and head nurses; not implemented
		Agreement on transfusion process in ED observation unit	HCM#3 / SCM#2	Discussed among attending physicians; completed
Organization		Meeting with ICU representatives and revision of patient transfer agreements from ED observation unit	HCM#3 / SCM#2	Not implemented
Organization	High pressure environment - point of triage (i.e., poor and narrow design of triage area; understaffing; lack of qualified personnel for triage; ongoing project on redesign of triage process and assisting technology)	Repeated discussion of various solutions for point of triage in ED management meeting (with the objective to manage exceeding work load during triage)	HCM#4, HCM#5, HCM#7 / SCM#3, SCM#4	Few completed (e.g., blocking of external phone calls); but most solutions considered not feasible (e.g., separate room, free of distractions, permanent staffing of two qualified nurses at triage)
	Leadership (e.g., staff’s need for enhanced participation in meetings and ongoing reorganization)	External moderator for ED nursing staff meetings to allow for enhanced discussion and opportunities to ask questions	HCM#6 / SCM#3, SCM#4	Agreed, but not implemented at follow-up
		Ad hoc meeting for ED providers concerning reorganization of triage process	HCM#6 / SCM#3, SCM#4	Completed
	Lack of staff information (i.e., lack of status information concerning ongoing projects and reorganization activities in the ED)	Provision of Q&A sheet on reorganization of triage process for nurses in intranet	HCM#6 / SCM#3, SCM#4	Completed
	Staff shortages (i.e., permanent understaffing, particularly during times of high patient load)	Schedule additional nursing and medical providers in shifts	HCM#8 / SCM#4	Not implemented
		Realistic HR planning of ED personnel and shift staffing levels of ED nurses and physicians	HCM#8 / SCM#4	Not started at follow-up

*Legend*. *ED* emergency department, *SEIPS* Systems Engineering Initiative for Patient Safety model, *ICU* intensive care unit. #HCM / #SCM: Number of health circle meeting (HCM) or steering committee meeting (SCM), issue being discussed, analyzed, or reconsidered (HCM#1: feedback session of baseline results and development of action plan; SCM#1: feedback session of baseline results)

### Sample description

At baseline, 170 provider surveys were distributed whereof 76 were returned (response rate: 44.7%). At follow-up, 73 out of 157 surveys were returned (46.5%). Forty-one ED providers participated at both waves (29 ED nurses, 5 ED physicians, 7 ED administrators). Tests for panel attrition (between those who returned a complete survey both times and those who only answered at baseline or follow-up) indicated that the final sample reported higher professional tenure, higher ratings of work overload, and higher depersonalization (see Additional file 1: Table S2).

Overall, 160 observation sessions (80 each at baseline and follow-up) were conducted, resulting in 240 observation hours: 99 with ED nurses and 61 with ED physicians. One hundred fifty-six work stress reports were collected after observations (76 at baseline, 80 at follow-up).

Altogether, 1418 ED patients were surveyed; 694 at baseline (survey response rate: 69.2%) and 724 at follow-up (81.2%).

### Changes in work system factors

At baseline, respondents reported high levels of patient stressors, work overload, information problems, and uncertainty. Participation opportunities, personnel resources, and supervisor feedback were rated as below average (see Table 2). Considering mean changes over time, job control significantly increased (*p* = 0.01). Mean weekly overtime significantly decreased from 7.3 to 5.8 h (*p* = 0.01). Supervisor feedback improved although this change was not significant (*p* = .058). However, social support deteriorated at follow-up (*p* < 0.01). Considering Cohen’s delta, effect sizes for changes in work factors were rather weak ranging from Δ = .31 (social support) to Δ = .42 (overtime) (see Table 2).

**Table 2:** Descriptive statistics and changes in work factors, respondent well-being, and quality of care (provider survey)

Study outcomes	Scale range	No. of items	Baseline (T1)		Follow-Up (T2)		Effect size	t-test		
			Mean	SD	Mean	SD	(Cohen’s d)	t	df	p
Work system factors										
Patient stressors	1–5	3	4.10	.63	4.11	.71	.02	−.10	.40	.921
Job control	1–5	4	2.63	.76	2.90	.74	.36	−2.57	39	**.014**
Participation opportunities	1–5	4	1.81	.70	1.83	.70	.03	−.24	39	.813
Work overload	1–5	3	4.37	.61	4.37	.48	–	.08	40	.937
Personnel resources	1–5	2	1.93	.70	1.76	.75	.23	1.13	39	.265
Information problems	1–5	3	3.20	.79	3.26	.80	.08	−.55	40	.583
Uncertainty	1–5	4	3.46	.65	3.64	.61	.29	−1.73	39	.091
Overtime (in hours)	–	1	7.79	3.93	5.93	4.92	.42	3.00	13	**.010**
Social support	1–5	2	3.15	.87	2.88	.89	.31	3.27	40	**.002**
Supervisor feedback	1–5	2	2.05	.90	2.30	.93	.27	−1.96	39	.058
Provider well-being										
Emotional exhaustion	1–6	4	4.19	.94	4.21	1.03	.02	−.18	40	.855
Depersonalization	1–6	4	3.18	1.23	3.54	1.22	.29	−2.29	40	**.027**
Depressive symptoms	1–4	2	1.90	1.48	2.22	1.53	.21	−1.59	40	.119
Job satisfaction	1–7	1	4.42	1.24	3.79	1.49	.46	2.60	37	**.013**
Turnover intentions	1–5	1	2.29	1.01	2.72	1.28	.37	−3.12	40	**.003**
Quality of care (ED provider reports)										
Frequency of errors	1–5	3	1.98	.74	2.05	.75	.09	−.54	40	.591
Patient safety	1–5	1	2.71	.78	2.50	.71	.28	1.90	40	.064

*Legend. N* = 41 participants; ED: emergency department, SD: standard deviation, d: delta, t: t-test statistic, df: degrees of freedom, p: probability level; bold if *p* < .05

No significant changes were observed in mean daily ED workload, workflow interruptions by patients, and respondents’ time spent in personal breaks (see Table 3). Intra-professional interruptions (e.g., nurse interrupts nurse) decreased before the intervention (β = − 0.1, *p* = 0.04), whereas inter-professional interruptions (e.g., nurse interrupts physician) significantly increased after the intervention (β = 0.1, *p* = 0.03). Mean interruptions by relatives decreased after the intervention (β = − 0.7, *p* = 0.03).

**Table 3:** Changes in day-level work factors, provider well-being, and patient reports of ED care (segmented regression analyses)

Study outcomes	ARIMA parameters										
	Intercept		Trend pre-intervention		Level change		Trend post- intervention		Workload		Goodness of fit (Rsq)
	β	*p*	β	*p*	β	*p*	β	*p*	β	*p*	
Work system factors											
Time spent in breaks (in %)	2.64	*.060*	−.01	*.834*	−.67	*.635*	.04	*.522*	−.14	*.181*	.12
Interruption rates by patients	.46	*.607*	−.01	*.588*	−.31	*.686*	.04	*.210*	.02	*.732*	.29
Interruption rates by relatives	.26	*.434*	−.02	*.067*	−.67	***.029***	.02	*.062*	.03	*.285*	.37
Interruption rates by colleagues of the same profession	3.84	***.007***	−.07	***.041***	1.04	*.334*	.06	*.181*	−.11	*.291*	.41
Interruption rates by colleagues of other ED professions	2.36	***.047***	−.05	*.103*	−.98	*.318*	.095	***.026***	.02	*.821*	.31
Provider well-being											
Work stress	1.36	***.002***	.01	*.288*	.57	*.127*	−.02	*.155*	.03	*.369*	.15
Quality of ED care (Patient reports)											
Overall satisfaction with ED care	1.66	***<.001***	.01	*.205*	.08	*.781*	−.02	*.197*	.056	***.016***	.33
Patient-oriented organization	3.24	***<.001***	−.01	*.132*	.08	*.658*	.02	***.022***	.000	*.980*	.67
Patient-oriented interaction	4.63	***<.001***	−.01	***.045***	.16	*.443*	.01	*.304*	−.03	*.093*	.30
Patient-oriented information	4.03	***<.001***	.00	*.727*	.03	*.866*	.01	*.545*	−.01	*.553*	.40
Satisfaction with waiting time	4.09	***<.001***	−.02	***.011***	−.06	*.778*	.03	***.011***	−.04	*.072*	.48

*Legend*. ARIMA: Autoregressive integrated moving average, Rsq: R-square, β: standardized regression coefficient, bolded if *p* < .05

### Changes in respondents’ well-being

At baseline, 61% of respondents reported high emotional exhaustion and 22% depressive symptoms above cut-off. At follow-up, the proportion of ED respondents with reported emotional exhaustion (75.6%) and depressive symptoms increased (34.1%). Both trends were not statistically significant. Depersonalization significantly increased over time (*p* = 0.01; see Table 2). Respondents further reported less job satisfaction (*p* = 0.01) and higher turnover intentions at follow-up (*p* < 0.01). However, mean daily work stress did not change significantly (see Table 3).

### Changes in quality of care

Respondents’ reports of the frequency of medical errors and overall ED patient safety remained stable over time (see Table 2).

However, significant changes in patient-perceived quality of care were observed (see Table 3). At follow-up, patient evaluations of ED organization improved (β = 0.02, *p* = 0.02). Further, ratings of waiting time declined before the intervention (β = − 0.02, *p* = 0.01), but improved significantly after the intervention (β = 0.03, p = 0.01). Concerning interaction with ED providers, a negative pre-intervention trend was observed (β = − 0.01, *p* = 0.045), however, no significant subsequent changes were identified. Patient’s overall satisfaction with ED care remained stable at a high level. Daily ED workload negatively predicted overall patient satisfaction (β = 0.1, p = 0.02), such that patients were less satisfied with overall quality of care on days with less favorable patient/provider-ratios.

### Evaluation of intervention fidelity and implementation process

Forty-one surveys were collected after health circle meetings. Participants indicated high satisfaction with meeting outputs (mean, M = 4.2; standard deviation, SD = 1.0), meeting atmosphere (M = 4.7, SD = 0.5), and opportunities to actively develop solutions for work problems (M = 4.1, SD = 0.7). Participants also reported high motivation to improve work conditions (M = 4.0, SD = 1.0). However, developed measures were deemed only partially realizable in everyday work (M = 3.3, SD = 1.1).

#### Implementation process

Stakeholder interviews revealed that provider surveys and work observation sessions addressed relevant aspects of the ED environment and that sufficient information was available prior to the intervention. Despite extensive staff information and communication efforts prior to study start, some ED providers felt not well informed about the project purpose and process; among interviewees, two out of nine respondents did not take full note of surveys and expressed limited time capacities to deal with available information.

#### Contextual factors and mental models

Nurses’ intervention participation was deemed successful while there was concern about lack of physician involvement. Two interviewees explicitly mentioned the intervention’s potential to improve ED work conditions. However, only two respondents expressed motivation to actively engage in improvement activities. Few expressed reservations that implementation of measures might be difficult due to organizational constraints (e.g., shortage of personnel resources). Main stakeholder expectations concerning the study were to raise awareness for ED workload in other wards and at hospital management level.

#### Intervention effects

When asked about changes in their work environment, three interviewees reported a deterioration of their work situation, while others reported no changes or slight improvements, especially relating to personal breaks. Three respondents reported a general increase in work stressors (i.e., increased patient numbers, insufficient staffing). Nonetheless, after study completion, the hospital’s health promotion department decided to roll out the intervention to other hospital units. This was partly motivated by the overall positive feedback of ED providers concerning the participatory approach and involvement of representatives from the worker’s council. Moreover, health circle meetings were considered a feasible opportunity for the evaluation of psychosocial risk factors at work.

## Discussion

To the best of our knowledge, this study is the first to systematically investigate prospective effects of a multi-professional organizational-level intervention on ED work conditions, provider well-being, and quality of care. Patient perceptions of ED organization and waiting times, and survey respondents’ self-reported job control and overtime hours improved while some indicators of provider well-being deteriorated. Given the lack of organizational-level intervention research in EDs [2], our results generate first valuable insights into the feasibility and effects of participatory interventions on ED provider and patient outcomes.

Theoretical assumptions of our study were based on the SEIPS model which links multiple factors of the work system with care processes and provider and patient outcomes [13, 14]. However, observed intervention effects in our study were inconsistent across different outcomes. Considering work factors, *job control* is a key resource for provider well-being and performance [1, 2]. Participatory interventions were shown to increase job autonomy partly due to their inclusive approach and employee-oriented focus [15, 20]. Our results confirm this assumption for the ED context since survey respondents reported significantly higher job control at follow-up. Furthermore, *workflow interruptions* are a major work stressor in EDs [29, 41]. In our study, inter-professional interruptions increased after the intervention, which suggests more face-to-face communication and information exchange across professions. Yet, we cannot infer about the underlying reasons and consequences of this increase, e.g., if additional interruptions were more helpful or necessary. Further, interruptions by relatives decreased which might indicate better information of patients and relatives about ED procedures resulting in less need to interrupt ED providers. Accordingly, a key finding of our study was that patients reported significant improvements in *ED organization and waiting times*. Generally, EDs are interrupt-driven environments and excessive interruptions mitigate provider well-being and performance [6, 42, 43]. However, disruptions can also contribute to efficient and timely patient care [6, 44]. Our results hint to this double-edged sword: frequent interruptions among providers may promote patient-perceived ED organization and shorter waiting times but also contribute to inferior provider well-being.

Our results further corroborate that *burnout* is a chronic work-related hazard of the ED work environment that affects a significant proportion of ED physicians and nurses [2–4]. In our study, job satisfaction decreased while turnover intentions and depersonalization increased at follow-up. Available evidence on effects of organizational interventions on healthcare provider mental well-being is inconsistent [18, 45]. Based on stakeholder interviews, we assume that observed deteriorations in well-being were related to provider’s disappointment about shortcomings in the implementation of developed measures [46]. Furthermore, mental well-being might have affected ED providers’ willingness to engage with intervention measures [18]. Low job satisfaction and low affective well-being were shown to predict intervention participation and evaluation of intervention effectiveness in elder care providers [47]. Moreover, since duration of personal breaks was unchanged over time, opportunities for respite or recovery from high work strain during ED shifts remained limited [30].

Finally, an unexpected study finding was that *social support* from colleagues decreased at follow-up. Designing sociotechnical work systems which promote effective teamwork is crucial for positive provider and patient outcomes [14]. However, in our study, ED providers reported concerns that initiated measures were not pursued due to resource limitations, institutional boundaries, and organizational constraints, i.e., financial cuts and a long-standing surge in patient load. Adverse contextual conditions as well as insufficient support by colleagues and managers in implementing measures might thus have led to disappointment and decreased trust within the ED team [17, 36, 48]. Moreover, those interventions with the highest progress of implementation at follow-up were related to improving communication and organization between ED providers, while solutions that involved more resources (i.e., personnel) were among those that were not (or not yet) implemented. Despite consistent participation and support of ED management throughout the study, providers may have developed a perception of limited support since effects did not exert on the ED organizational level as anticipated [17, 36].

### Limitations

We established a mixed-methods ITS study design that allows robust inferences concerning prospective changes of outcomes between pre- and post-intervention assessments [21]. However, pragmatic improvement studies in dynamic clinical settings comprise multiple limitations. First, although our study setting features a typical urban ED setting and relies on elaborated analyses, it lacks a control group. This limits inferences concerning causation as well as secular trends. We describe a realist approach that aimed to change ED work factors. This comprehensive approach targeting several task- and organizational-level aspects over one year does not allow for attribution of effects to single interventions or steps. Our participatory approach consists of several interventions of collective intertwined initiatives that occur in the course of a multitude of everyday concurrent events in patient care [17]. Therefore, we cannot attribute effects of specific measures to primary and secondary outcomes nor specify time lags of measures being effective. Second, our results strongly depend on local contextual factors and the process of intervention implementation [35]. In the study period, other process changes occurred, i.e., reorganization of the triage process and preparation of constructional expansion. ED providers thus might have perceived limited capacity to engage with intervention measures on top of high daily workloads. Nevertheless, our approach combined quantitative results with qualitative information from stakeholder interviews to shed light on these potentially relevant facilitators and barriers in intervention implementation [36, 46]. Third, we acknowledge the rather small longitudinal sample of provider surveys. Although high commitment in data collection and recruitment was undertaken, follow-up bias occurred. This might partially be due to high staff turnover rates which are generally characteristic of EDs [3]. To offset limitations of provider ratings which are prone to subjective bias, we further used objective and independent methods such as expert observation sessions, patient surveys, and register data to measure study outcomes. Yet, observations were confined to day-time shifts which limit inferences concerning provider workflow routines during evening or night shifts [49]. The patient survey tool has been previously applied in various populations including ED patients where it proved its reliability [6]. Specific investigations into the validity of the instrument for ED patient surveys are not yet reported. Fourth, our timing of follow-up assessment needs careful consideration [50]. For practical reasons, we used a one-year time lag. However, ongoing improvements might have failed to reach their full impact on work system factors and provider well-being at the time of our follow-up measurement [17, 51]. Finally, we acknowledge that ED work systems comprise multiple factors and that system interventions should comprehensively address various components to improve provider and patient outcomes [13, 17, 42, 52]. Yet, although preliminary evidence points to positive effects of comprehensive organizational-level interventions for provider outcomes [17], simultaneous effects for patient care need to be elicited. In our study, solutions concerning other domains such as changes in technologies, tools, or environmental factors, were considered by ED providers in meetings but not prioritized for implementation. This refers particularly to resource constraints such as understaffing or structural provisions of the physical environment. Although we carefully introduced the intervention approach to providers and sought to manage stakeholder expectations in the beginning of the study, post-hoc, we cannot infer on specific anticipations or ‘implicit theories’ [36] of involved stakeholders, e.g., employee assumptions that hospital management would provide additional resources or prioritization of staff shortages.

### Implications for practice and further research

Implementation of organizational-level interventions is time-consuming and evaluation of intervention effects is challenging [53]. We used a multi-disciplinary intervention that included both ED nurses and physicians in collaborative meetings and implementation of solutions. Yet, this partly resulted in perceptions of imbalanced involvement, efforts, and contributions of both professions. Future attempts should therefore seek opportunities to implement interventions that take account of the multi-disciplinary nature of ED work as well as consider unique expectations and needs of each profession in the course of participatory work design in clinical care [3]. Concerning methodological aspects, future studies should consider applying cluster-randomized and controlled designs across various ED settings as well as realist evaluation for intervention evaluation [46]. Varying follow-up measurement intervals should be considered to capture potentially time-delayed intervention effects [50]. With regard to intervention content, future studies should expand the scope of assessed work system factors as well as their differential effects on processes, provider and patient outcomes [14]. Furthermore, ED practitioners could expand or adapt existing tools and practices in performance management to include continuous improvement of work system factors [18]. Finally, although our intervention approach was well-accepted by ED providers, partial improvements in work conditions and patient evaluations of care were accompanied by deteriorations in provider mental well-being. Future studies of work system interventions should thus elucidate beneficial concomitants of provider well-being before and during intervention implementation in high stress care environments.

## Conclusions

This study provides valuable first insights into the feasibility of organizational-level interventions in EDs to improve work conditions, provider well-being, and quality of care. Our findings indicate that interprofessional approaches targeting work system factors are well-accepted by ED physicians and nurses. Improvement measures developed by ED providers largely focused on changes in organizational work factors. Improvements in job control, overtime hours, and patient perceptions of ED organization and waiting times were observed. However, provider well-being deteriorated over time. Future studies should further identify to what extent and under which circumstances work system interventions are beneficial for provider outcomes in high stress care environments.

## Additional file

Additional file 1: **Table S1.** Dates of data collection. Dates of data collection at baseline and at follow-up. **Table S2** Panel attrition: Loss to follow-up analysis. Analysis of loss to follow-up in provider survey participants from baseline to follow-up. (PDF 36 kb)

## Abbreviations

ARIMA: Autoregressive integrated moving average
ED: Emergency department
ESI: Emergency Severity Index
ITS: Interrupted time-series
SEIPS: Systems Engineering Initiative for Patient Safety
