# Supplementary material for: Effects of work conditions on provider mental well-being and quality of care: a mixed-methods intervention study in the emergency department
Source: BMC Emerg Med. 2019 Jan 3;19:1. doi: 10.1186/s12873-018-0218-x (PMC6318954; doi:10.1186/s12873-018-0218-x)
Supplement: Supplementary file 1 — Table S1. Dates of data collection. Dates of data collection at baseline and at follow-up. Table S2 Panel attrition: Loss to follow-up analysis. Analysis of loss to follow-up in provider survey participants from baseline to follow-up. (PDF 36 kb) [file 12873_2018_218_MOESM1_ESM.pdf]

Supplementary Tables S1 and S2

Table S1: Dates of data collection

Day number	Date: Baseline	Date: Follow-up
1	01.12.2015	02.12.2016
2	02.12.2015	06.12.2016
3	07.12.2015	07.12.2016
4	09.12.2015	08.12.2016
5	08.12.2015	12.12.2016
6	10.12.2015	14.12.2016
7	14.12.2015	16.12.2016
8	18.12.2015	18.12.2016
9	19.12.2015	20.12.2016
10	20.12.2015	22.12.2016
11	04.01.2016	09.01.2017
12	07.01.2016	11.01.2017
13	08.01.2016	13.01.2017
14	12.01.2016	17.01.2017
15	13.01.2016	19.01.2017
16	14.01.2016	21.01.2017
17	15.01.2016	23.01.2017
18	18.01.2016	25.01.2017
19	20.01.2016	27.01.2017
20	22.01.2016	30.01.2017

Table S2: Panel attrition

Study outcomes	Full sample T1 and T2 (n=41)	Dropouts T1 not T2 (n=35)	Mean differences at baseline (T1)			Full sample T1 and T2 (n=41)	"Dropins" T2 not T1 (n=32)	Mean differences at follow-up (T2)		
	M (SD) T1	M (SD) T1	t	df	p	M (SD) T2	M (SD) T2	t	df	p
Work system factors										
Professional tenure	21.22 (11.30)	13.06 (9.22)	-3.28	68	**.002**	21.09 (12.02)	11.41 (10.92)	-3.55	71	**.001**
Patient stressors	4.10 (.63)	4.03 (.79)	-.42	74	.673	4.11 (.71)	4.13 (.59)	.12	71	.902
Job control	2.61 (.76)	2.93 (.88)	1.69	74	.095	2.90 (.74)	2.85 (.76)	-.30	70	.763
Participation opportunities	1.80 (.70)	2.01 (.69)	1.31	74	.196	1.83 (.70)	1.93 (.61)	.63	70	.532
Work overload	4.37 (.61)	4.07 (.83)	-1.81	61	.075	4.37 (.48)	4.04 (.67)	-2.41	71	**.018**
Personnel resources	1.90 (.71)	2.13 (.63)	1.45	74	.150	1.76 (.75)	1.90 (.72)	.78	70	.441
Information problems	3.20 (.79)	3.15 (.75)	-.29	74	.776	3.26 (.80)	3.11 (.80)	-.77	71	.444
Uncertainty	3.48 (.65)	3.58 (.69)	.67	74	.505	3.64 (.61)	3.42 (.56)	-1.53	70	.132
Overtime (hours)	7.26 (4.78)	9.84 (10.32)	1.14	33	.262	5.81 (5.10)	7.84 (9.59)	.83	35	.411
Social support	3.15 (.87)	3.33 (1.05)	.83	74	.410	2.88 (.89)	3.20 (.98)	1.48	71	.144
Supervisor feedback	2.02 (.90)	2.46 (1.15)	1.80	64	.077	2.30 (.93)	2.41 (.91)	.49	70	.629
Provider well-being										
Job satisfaction	4.35 (1.25)	4.62 (1.48)	.84	72	.402	3.79 (1.47)	4.40 (1.45)	1.70	67	.093
Turnover intentions	2.29 (1.01)	2.06 (1.03)	-1.01	74	.317	2.72 (1.28)	2.16 (1.14)	-1.96	71	.054
Emotional exhaustion	4.19 (.94)	3.96 (1.19)	-.91	74	.366	4.21 (1.03)	3.88 (1.18)	-1.25	71	.214
Depersonalization	3.18 (1.23)	3.03 (1.29)	-.53	74	.600	3.54 (1.22)	2.87 (.93)	-2.57	71	**.012**
Depressive symptoms	1.95 (.74)	1.90 (.74)	-.30	74	.764	2.11 (.76)	2.08 (.87)	-.15	70	.880
Quality of care										
Frequency of errors	1.98 (.12)	1.69 (.52)	-2.02	71	.047	2.05 (.75)	2.09 (.70)	.26	71	.795
Patient safety	2.71 (.78)	3.04 (.67)	1.99	74	.050	2.50 (.71)	2.81 (.82)	1.75	71	.085

Legend. T1: baseline, T2: follow-up; M: mean, SD: standard deviation, t: t-test statistic, df: degrees of freedom, bolded if p < .05.
